# Anti-adhesion Property of the Potential Probiotic Strain *Lactobacillus fermentum* 8711 Against Methicillin-Resistant *Staphylococcus aureus* (MRSA)

**DOI:** 10.3389/fmicb.2018.00411

**Published:** 2018-03-08

**Authors:** Sathyanarayanan Jayashree, Raman Karthikeyan, Sampath Nithyalakshmi, Jothi Ranjani, Paramasamy Gunasekaran, Jeyaprakash Rajendhran

**Affiliations:** ^1^Department of Genetics, School of Biological Sciences, Madurai Kamaraj University, Madurai, India; ^2^VIT Bhopal University, Sehore, India

**Keywords:** MRSA, Caco-2 cells, *Lactobacillus fermentum*, cytotoxicity, probiotics, anti-adhesion

## Abstract

Methicillin-resistant *Staphylococcus aureus* (MRSA) is a multidrug-resistant pathogen and one of the leading causes of nosocomial infection worldwide. Probiotic bacteria play a significant role in preventive or therapeutic interventions of gastrointestinal infections in human as well as animals. In this study, we have investigated the adhesion property of the probiotic strain *Lactobacillus fermentum* MTCC 8711 and its ability to prevent the adhesion of MRSA to human colon adenocarcinoma cells, Caco-2. We have shown that *L. fermentum* could efficiently adhere to the Caco-2 cells. Also, we have shown that *L. fermentum* significantly reduced MRSA adhesion to Caco-2 cells. Three types of experiments were performed to assess the anti-adhesion property of *L. fermentum* against MRSA. Inhibition (Caco-2 cells were pre-treated with *L. fermentum*, and subsequently MRSA was added), competition (both *L. fermentum* and MRSA were added to Caco-2 cells simultaneously), and displacement or exclusion (Caco-2 cells were pre-treated with MRSA, and subsequently *L. fermentum* was added). In all three experiments, adhesion of MRSA was significantly reduced. Interestingly, *L. fermentum* could efficiently displace the adhered MRSA, and hence this probiotic can be used for therapeutic applications also. In cytotoxicity assay, we found that *L. fermentum per se* was not cytotoxic, and also significantly reduced the MRSA-induced cytotoxicity. The protective effect occurred without affecting Caco-2 cell morphology and viability.

## Introduction

Methicillin-resistant *Staphylococcus aureus* (MRSA) is one of the leading causes of healthcare- and community-associated infections (Naimi et al., 2003). Decades of research have illuminated how this organism has evolved with virulence factors that contribute to the diversity and severity of staphylococcal infections. *S.* *aureus* efficiently colonizes on the skin surface, intestinal tract and mucous membranes of the host with mild clinical features, and it is estimated that 20% of the world’s population are persistent carriers (Sollid et al., 2014). It is the most common cause of infections in skin and soft tissue, and once it reaches the blood through subcutaneous tissues, it can infect almost any organ, most notably bone tissue and cardiac valves. Community-associated MRSA strains colonize the intestinal tract of humans in particular among the hospitalized patients and infants who have had prolonged hospital stays. Clinical manifestation of MRSA includes necrotizing pneumonia, necrotizing fasciitis, pyomyositis, skin and soft tissue infections. MRSA was considered primarily as a healthcare-associated pathogen, but recent findings suggested that MRSA can infect through contaminated foods as well. A recent survey indicated the presence of MRSA in food products obtained from retail markets in the United Kingdom (Hadjirin et al., 2015). Timothy et al. (2002), community-acquired MRSA gastroenteritis outbreak was recorded in the United States. The outbreak-related MRSA strains were reported to produce enterotoxins A, C, or D, responsible for the gastrointestinal illness.

Development of multidrug resistance among MRSA strains poses a significant challenge to successful treatment. Also, antibiotic treatment disrupts the normal microbiota of the gut, which in turn makes serious complications in the absorption and ingestion of nutrients from the diet. Since MRSA resides in the normal microflora, it could not be eliminated easily with antibiotics. Therefore, interventions using probiotics have a strong preventive and therapeutic value in the management of MRSA infection. “Probiotics” are live microorganisms which when administrated in adequate amounts confer a health benefit on the host (FAO/WHO, 2001; Schrezenmeir and De Vrese, 2001). Most commonly used probiotic bacteria include *Lactobacillus* and *Bifidobacterium*. They are known to resist gastric acid, bile salts, and pancreatic enzymes, and to adhere to colonic mucosa and readily colonize the intestinal tract (Lu and Walker, 2001). Lactobacilli are an important part of the indigenous microbiota of man and higher animals. Probiotics are usually taken after antibiotic therapy to restore the beneficial microbial population. However, regular consumption of probiotic microorganisms will be useful to establish a positive balance of the population of beneficial microbes in the intestinal microbiota (Cox et al., 2010; Bruzzese et al., 2014).

Probiotics modulate the indigenous intestinal microbiota and improve the health via multiple mechanisms, including the inhibition of enteric pathogens by decreasing the gut pH, secretion of bacteriocins and the stimulation of defensin production by the host epithelial cells. They may also inhibit the pathogen attachment and subsequent invasion of epithelial cells by competing for surface receptors, a process called as colonization resistance (Stecher and Hardt, 2011). Hence, the probiotics can be used to control the growth of pathogens and thereby control and/or prevent infections. Lactic acid bacteria (LAB) represents the major group of probiotic bacteria and *Lactobacillus* represents the dominant genus of the LAB. *Lactobacillus* is a Gram-positive, facultative anaerobic or microaerophilic bacteria that represent a significant portion of the human microbiota (Walter, 2008). Several species of *Lactobacillus* have been shown to promote health in human and animals (Kumar et al., 2015). *Lactobacillus fermentum* is a heterofermentative LAB considered as a potential probiotic bacterium widely found in dairy products such as milk, yogurt, and cheese (Pereira et al., 2003). It inhabits the human gastrointestinal and urogenital tracts. It has the GRAS (Generally Regarded As Safe) status, and possess beneficial properties such immunomodulation, cholesterol reduction, reduction in severity of symptoms caused by upper and lower tract respiratory illnesses and gastrointestinal infections (West et al., 2011). *L. fermentum* is well-known to survive in the gastric transit, adhere to the intestinal epithelial cells, extracellular matrix and in some cases known to colonize and persist in the gut. *L. fermentum* MTCC 8711 was isolated from yogurt, and it has potential probiotic properties such as acid tolerance, bile tolerance, and β-galactosidase activity (Jayashree et al., 2010). The ability of a probiotic bacterium to adhere to the epithelial cells of the intestinal tract is a prerequisite for establishing colonization. Efficient colonization of *L. fermentum* could be responsible for the inhibition and exclusion of gastroenteric pathogens. Bacterial adhesion ability to the human epithelial cells can be assessed with *in vitro* adhesion assay using Caco-2 cells. This colon carcinoma cells on full differentiation have characteristics of mature enterocytes with functional brush border microvilli and apical hydrolases. Thus, it has become a routine model for performing bacterial adhesion and invasion experiments. In this study, we evaluated the adhesion property of *L. fermentum* 8711 and its anti-adhesion property against MRSA in Caco-2 cells.

## Materials and Methods

### Bacterial Strains and Growth Conditions

*Lactobacillus fermentum* MTCC 8711 and MRSA ATCC 43300 were grown in de Man, Rogosa and Sharpe (MRS) broth (pH 6.5) (Himedia, Mumbai, India) and brain heart infusion (BHI) broth (pH 7.4) (Himedia), respectively at 37°C. Both strains were maintained at -80°C in the appropriate cultivation broth containing 20% (v/v) glycerol.

### Cultured Cell Lines

The Caco-2 human colon adenocarcinoma cell line was obtained from the American Type Culture Collection. Cells were routinely maintained in DMEM F12 Ham (pH 7.6) (Himedia) supplemented with non-essential amino acids, L-glutamine, sodium pyruvate, antibiotics, and 20% of heat-inactivated FBS at 37°C in a humidified chamber with 5% CO_2_ supply. The cryopreserved cells were thawed rapidly by placing the vial at 37°C. Cells were transferred to a T_25_ flask containing 5 ml of pre-warmed complete culture media. The cells were seeded at a density of 4 × 10^5^ cells/ml. The cells were grown at 37°C in a 95% air-5% CO_2_ for 10–15 days till they form a monolayer with 70% confluency. At 70–80% confluence, cells were trypsinized for 3–5 min. Complete culture medium was added to each flask and mixed with the trypsinized cells to inactivate trypsin. Cells were centrifuged at 1000 rpm for 2 min at room temperature in a 15-ml centrifuge tube. The supernatant was removed, and the pellet was resuspended into 8 ml of culture medium. Following the cell counts by trypan blue exclusion, subsequently, cells were subcultured into T25/T75 flasks. Media was replaced routinely every 2 days.

### Assessment of Adhesion Property of *L. fermentum* 8711 on Caco-2 Cells

The adhesive property of *L. fermentum* MTCC 8711 was evaluated using the Caco-2 cell line model. *In vitro* adhesion assays were performed as described by Bouchard et al. (2013). Caco-2 monolayer cells were prepared in 6-well plates, washed twice with phosphate-buffered saline (PBS, pH 7.4) to remove the unattached cells. An overnight culture (16 h) of *L. fermentum* MTCC 8711 (10^7^ CFU/ml) was harvested and co-cultured with the monolayer of Caco-2 cells for 1 h at 37°C in antibiotic- and serum-free DMEM-F12 HAM. After 1 h of incubation, the monolayers were washed three times with sterile PBS to remove non-adherent bacteria. The adhered bacteria were detached by adding 1 ml of 0.04% Tween 80, serially diluted with PBS, and appropriated dilution was plated onto MRS agar plates. The CFUs were calculated after overnight growth at 37°C to determine the number of adhered bacteria on Caco-2 cell monolayer.

For microscopic observation, an overnight culture of *L. fermentum* 8711 grown in MRS broth was pelleted and suspended in 1 ml of 0.1 M sodium carbonate buffer. Fluorescein isothiocyanate (FITC) at a final concentration of 2 μg/ml was added to the suspension and incubated at 20°C for 60 min. After incubation, the bacterial cells were washed three times with PBS to remove the excess stain and suspended in 1 ml of antibiotic-free DMEM. The Caco-2 cells were stained with Mitotracker Deep Red (1 μg/ml) and Hoechst (1 μg/ml), incubated for 30 min at 37°C in a 5% CO_2_. Bacterial suspension (*L. fermentum*) was added with MOI of 1:100 to Caco-2 cells monolayer. The plate was incubated for 1 h at 37°C in 5% CO_2_–95% air. The cells were washed twice with PBS. The cells were carefully fixed to a glass slide using methanol and glycerol. The fixed cells were observed, and images were captured using high content screening system-Operetta (Perkin Elmer).

Also, adhesion efficiency of *L. fermentum* was evaluated by flow cytometry analysis. Briefly, Caco-2 cells were grown in 6-well plates containing complete culture medium at 37°C with 5% CO_2_ until they reach 70% confluency. After achieving the confluency, cells were harvested by trypsinization and centrifuged at 1500 rpm for 5 min. Then the Caco-2 cells were stained with Mitotracker Deep red and subsequently treated with FITC stained *L. fermentum* and incubated for 1 h. After incubation, treated cells were centrifuged at 1000 rpm for 2 min and washed twice and resuspended in sterile PBS and subjected to flow cytometry analysis using FACSAria III (Becton Dickinson, San Jose, CA, United States).

### Assessment of Adhesion Property of MRSA on Caco-2 Cells

The adhesion property of MRSA was evaluated using Caco-2 cell line. Caco-2 monolayer cells were prepared in 6-well plates, washed twice with phosphate-buffered saline (PBS, pH 7.4) to remove the unattached cells. An overnight culture (16 h) of MRSA was harvested and infected with the monolayer of Caco-2 cells for 1 h at 37°C in antibiotic and serum free DMEM-F12 HAM. After 1 h of incubation, the monolayers were washed three times with sterile PBS to remove unbound bacteria. The adhered bacteria were detached by adding 0.1X Triton-X, serially diluted with PBS, and appropriated dilution was plated on Mannitol Salt Agar agar (pH 7.4) (Himedia). The plates were incubated overnight at 37°C to determine the number of adhered bacteria on Caco-2 cell monolayer.

For microscopic observation, an overnight culture of MRSA grown in BHI broth was centrifuged, and the cells were suspended in 0.1 M sodium bicarbonate buffer. The bacterial suspension was stained with FITC (25 μg/ml) for 30 min at 37°C. The Caco-2 cells monolayer was stained with Mitotracker Deep Red (1 μg/ml) and Hoechst (1 μg/ml), incubated for 30 min at 37°C in a 5% CO_2_. MRSA with MOI of 1:10 was incubated with Caco-2 cells for 2 h at 37°C in a 5% CO_2_. After incubation, the cell monolayer was washed twice with PBS. Cells were observed and examined using high content screening (Operetta- Perkin Elmer).

Also, adhesion efficiency of MRSA was analyzed by flow cytometry. Caco-2 cells were grown in 6-well plates containing complete culture medium at 37°C with 5% CO_2_ until they reach 70% confluency. After achieving the confluency, cells were trypsinized and centrifuged at 1500 rpm for 5 min. Then, the Caco-2 cells were stained with Mitotracker Deep Red and hochest. Caco-2 cells were infected with acridine orange (AO) stained MRSA and incubated for 1 h. After incubation, infected cells were centrifuged at 1000 rpm for 2 min and washed and resuspended in sterile PBS and subjected flow cytometry analysis using FACSAria III.

### Anti-adhesion Property of *L. fermentum* Against MRSA on Caco-2 Cells

Anti-adhesion property *L. fermentum* against MRSA on Caco-2 cells was evaluated using three independent assays: (i) inhibition of adhesion, (ii) competitive adhesion, and (iii) displacement. For microscopic observation, the Caco-2 cells and MRSA were stained with Hoechst (1 μg/ml), and *L. fermentum* was stained with FITC and observed using high content screening system, Operetta (Perkin Elmer).

(1) Inhibition of adhesion of MRSA to Caco-2 cells by *L. fermentum*:

The adhesion inhibition assay was performed according to the method described by Gueimonde et al. (2006) with slight modifications. We used 1X PBS to wash the cells instead of HH buffer used by Gueimonde et al. (2006). The overnight culture of MRSA and *L. fermentum* MTCC 8711 were grown in TSB and MRS broth respectively. *L. fermentum* MTCC 8711 with MOI of 1:100 was incubated with Caco-2 monolayer separately for 1 h at 37°C in a 5% CO_2_–95% air. After incubation, unbound *L. fermentum* cells were removed by washing twice with 1X PBS followed by infection with MRSA (MOI 1:10) for 2 h at 37°C in a 5% CO_2_–95% air and number of bacteria adhering to the Caco-2 cells were determined by plating on MRS plates for *L. fermentum*, and MSA plates for MRSA after serial dilution followed by the incubation at 37°C for 24 h. Each experiment was repeated three times.

(1) Competitive adhesion assay:

The competitive adhesion assay was performed as described previously (Jankowska et al., 2008) with slight modifications. They have used the probiotic and pathogenic bacteria in equal numbers with MOI of 1:100. Based on the preliminary experimental results, we have used MRSA with MOI of 1:10 and *L. fermentum* with MOI of 1:100. The overnight culture of MRSA and *L. fermentum* MTCC 8711 were grown in their specific broth at 37°C. MRSA and *L. fermentum* cells were co-incubated with Caco-2 cells monolayer for 2 h at 37°C in a 5% CO_2_–95% air. After incubation, Caco-2 cells monolayer was washed twice with sterile PBS. Bacteria were detached from Caco-2 cells by Triton-X (0.1X) treatment, and the number of bacteria adhering to the Caco-2 cells were determined by plating on MRS and MSA plates and incubated at 37°C for 24 h.

(1) Displacement or exclusion assay:

An overnight culture of MRSA and *L. fermentum* MTCC 8711 were grown in their specific broth. MRSA with MOI of 1:10 was incubated with Caco-2 cells monolayer for 2 h at 37°C in a 5% CO_2_–95% air. After incubation, cells were washed twice with sterile PBS. The Caco-2 cells monolayers were then treated with *L. fermentum* MTCC 8711 with MOI of 1:100 and incubated further for 1 h under a 5% CO_2_ atmosphere. The cells were washed twice with PBS. Adhered bacteria were detached and serially diluted. Appropriate dilutions were plated in MRS and MSA plates and CFUs were estimated.

### MTT Cell Viability Assay

Caco-2 cells were seeded at a density of 10^5^ cells in a 12 well plate containing complete culture media and incubated at 37°C in a 95% air–5% CO_2_. The Caco-2 monolayer was washed twice with DMEM. The Caco-2 cells were infected with MRSA (10^6^ cells) for 2 h and incubated at 37°C in a 95% air–5% CO_2_. After incubation, the monolayer was washed twice with 1X PBS and 1 ml of complete culture media with 10 μg/ml lysostaphin was added. The plate was incubated at 37°C for 24 h. Subsequently, the Caco-2 cells monolayer was treated with *L. fermentum* with MOI of 1:100 in DMEM for 1 h at 37°C. After 1 h of incubation, the cell monolayer was washed twice with 1X PBS. The plate was incubated for 24 h at 37°C in a 95% air–5% CO_2_. Untreated Caco-2 cells were maintained as a control. After incubation, Caco-2 cells medium was removed from both MRSA, and *L. fermentum* treated cells and incubated in 0.5 mg/ml methylthiazolyldiphenyltetrazolium bromide (MTT) for 4 h at 37°C. After incubation, the solution was removed, and 500 μl of DMSO was added to dissolve the formazon crystals. Absorbance was read at 560 and 630 nm in Multimode Plate Reader (Perkin Elmer). Uninfected cells were used as a control. A subtraction analysis of the dual wavelength (D550 to D690) was performed to increase the accuracy of cytotoxicity measurement.

### Live-Dead Cell Assay Using FACS

Live and dead cell assay was performed to determine the viability of MRSA infected Caco-2 cells. Caco-2 cell monolayer was washed twice with 1X PBS, and the cells were suspended in DMEM medium. The cells were infected with MRSA at an MOI of 1:10 and further incubated for various time points (4, 16, and 24 h) at 37°C. At each time point, the cells were trypsinized using Trypsin-EDTA (HiMedia) for 3 min followed by addition of 20% FBS for inactivation. The trypsinized cells were centrifuged and suspended in 1X PBS. Propidium iodide (PI) (5 mg/ml) was added to each sample, and live/dead cells were quantified using FACSAria III. Uninfected cells were used as a control.

### Determination of MRSA-Induced Cytotoxicity in Caco-2 Cells After the Displacement by *L. fermentum*

Caco-2 cell monolayer was washed twice with 1X PBS and the cells were suspended in DMEM. The Caco-2 cell monolayer was infected with MRSA (MOI 1:10) and incubated at 37°C for 2 h with 5% CO_2_–95% air. After 2 h, the cells monolayer was washed and treated with *L. fermentum* (MOI 1:100) for 1 h at 37°C. After treatment; the cells monolayer was washed and incubated for 24 h at 37°C with 5% CO_2_–95% air. After incubation, the Caco-2 cells were trypsinized using Trypsin- EDTA (HiMedia) for 3 min and inactivated by adding 20% FBS. The trypsinized cells were centrifuged and suspended in 1X PBS. PI (5 mg/ml) was added to each sample, and cell viability was analyzed using FACSAria III.

### Statistical Analysis

All experiments were repeated three times, and the data are expressed as Mean ± SD. Data were analyzed for statistical significance using Student’s *t*-test and a *p*-value of <0.05 was used as a threshold for significance.

### Computational Prediction of Adhesins in the Genome of *L. fermentum*

*Lactobacillus fermentum* MTCC 8711 (Accession number: AVAB00000000) genome sequence was retrieved from NCBI. Adhesins of *L. fermentum* from the genome sequence were predicted using a Software program for Prediction of Adhesins and Adhesin-like proteins using Neural networks (SPAAN; Sachdeva et al., 2005). SPAAN uses neural networks integrated with five different attributes like amino acid frequencies, multiplet frequencies, dipeptide frequencies, charge composition, and hydrophobic composition. Proteins with P_ad_ (protein being an adhesin) score greater than 0.7 were considered as potential adhesins. Cellular localization of the potential adhesins was predicted using CELLO v.2.5 (Yu et al., 2006).

## Results

### Adhesion of *L. fermentum* 8711 and MRSA to Caco-2 Cells

**FIGURE 1:**
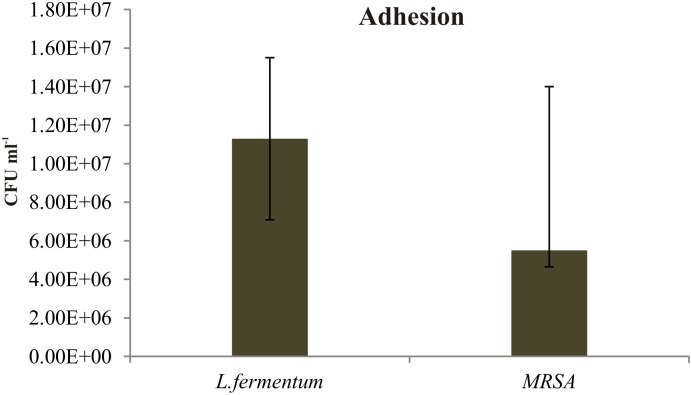
Adhesion of *Lactobacillus fermentum* and MRSA on Caco-2 cells. The adhesion of *L. fermentum* and MRSA were calculated by estimating the number of CFUs attached to the Caco-2 cells. The mean ± SD of three independent experiments are shown.

**FIGURE 2:**
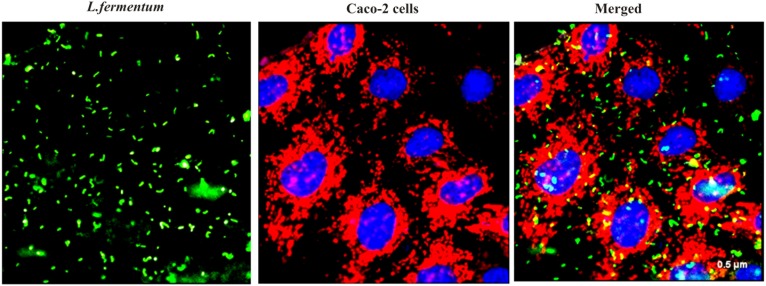
Adhesion of *L. fermentum* 8711 on Caco-2 cells after 2 h of incubation at 37°C in a 5% CO_2_ atmosphere *L. fermentum* was stained with FITC (Green fluorescence), and Caco-2 cells were stained with Hoechst and MTDR.

**FIGURE 3:**
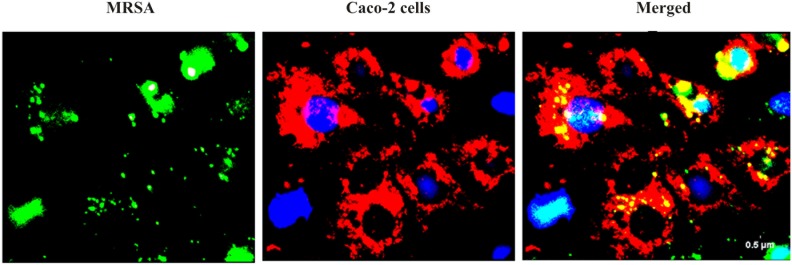
Adhesion of MRSA on Caco-2 cells after 2 h of incubation at 37°C in a 5% CO_2_ atmosphere. MRSA was stained with FITC (Green fluorescence), and Caco-2 cells were stained with Hoechst and MTDR.

**FIGURE 4:**
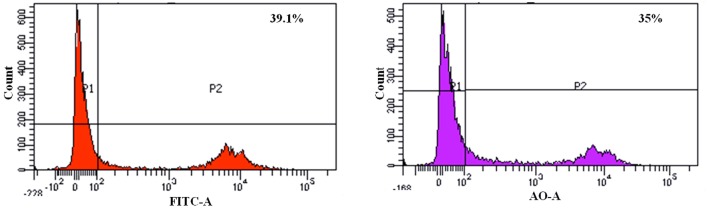
FACS analysis of adhesion of *L. fermentum* and MRSA on Caco-2 cells. (Left) Shows the adhesion of *L. fermentum* on 39.1% of Caco-2 cells. (Right) Shows the adhesion MRSA on 35% of Caco-2 cells.

We found that the *L. fermentum* 8711 has a potential adhesion property to the Caco-2 cells and the estimated adhered bacterial cells were 1.0 × 10^7^ CFU/ml. Similarly, we have studied the adhesion property of MRSA on Caco-2 cells and found that MRSA also potentially adhered to the surface of Caco-2 cells. CFU estimation revealed that the number of adhered MRSA cells was 4.7 × 10^6^ CFU/ml. Thus, comparatively, *L. fermentum* has stronger ability to adhere to Caco-2 cells than MRSA (**Figure 1**). **Figures 2**, **3** shows the microscopic observation of adhesion of FITC stained *L. fermentum* 8711 and MRSA, respectively, on the surface of Caco-2 cells. Flow cytometry analysis revealed that *L. fermentum* cells adhered to 39.1% of the Caco-2 cells and MRSA adhered to 35% of the Caco-2 cells (**Figure 4**).

### *L. fermentum* 8711 Inhibited the Adhesion of MRSA

Inhibition of adhesion, competition, and displacement assays were performed to evaluate the ability of *L. fermentum* to interfere with the adhesion of MRSA on Caco-2 cells.

### Inhibition of MRSA Adhesion by *L. fermentum*

**FIGURE 5:**
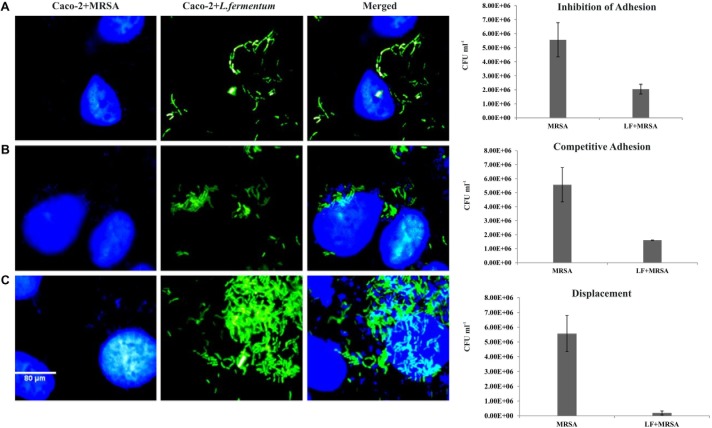
Effect of *L. fermentum* on the adhesion of MRSA to Caco-2 cells. Left panel: Microscopic observations: Caco-2 cells and MRSA were stained with Hoescht. Green fluorescence showed FITC stained *L. fermentum.* **(A)** Treatment of Caco-2 cells with *L. fermentum* followed by MRSA infection (Inhibition of adhesion). **(B)** Caco-2 cells were co-incubated with *L. fermentum* and MRSA (Competitive adhesion). **(C)** Infection of Caco-2 cells with MRSA followed by treatment with *L. fermentum*. Right panel: Estimated CFUs of adhered MRSA on three experiments. The CFU values are the average of three independent experiments with standard deviation.

Caco-2 cells were pre-incubated with *L. fermentum* for 2 h with the MOI of 1:100, followed by the addition of MRSA at an MOI of 1:10. The ability of *L. fermentum* to inhibit the adhesion of MRSA to Caco-2 cells is shown in **Figure 5A**. A significant reduction in the adhesion of MRSA was observed, and the mean CFU value (2.0 ± 0.35 × 10^6^) was 63.2% lower when compared to the treatment with MRSA alone. Further, we noted that the density of Caco-2 cell monolayer was not disturbed by MRSA as evident by direct microscopic examination and cell counting. Thus, *L. fermentum* 8711 could efficiently inhibit the adhesion of MRSA and protect the integrity of the Caco-2 cells.

### Competition Between *L. fermentum* and MRSA for Adhesion to Caco-2 Cells

We tested competitive adhesion of *L. fermentum* and MRSA on Caco-2 cells by co-incubation experiment. When *L. fermentum* was co-incubated with MRSA and Caco-2 cells for 2 h, adhesion of MRSA was significantly decreased with a CFU of 1.67 ± 0.13 × 10^6^ CFU/ml. The adhered CFU value was 71.1% lesser when compared to the MRSA infection alone (**Figure 5B**).

### Displacement of MRSA From Caco-2 Cells by *L. fermentum*

Pre-infection of Caco-2 cells with MRSA for 2 h and subsequent addition of *L. fermentum* resulted in an efficient displacement of adhered MRSA from the Caco-2 cells. The adhered CFU value of MRSA was 96.5% lesser when compared to the MRSA infection alone (**Figure 5C**). These results suggested that *L. fermentum* 8711 has potential anti-adhesion property against MRSA.

### Increased Viability of MRSA-Infected Caco-2 Cells Upon *L. fermentum* Treatment

**FIGURE 6:**
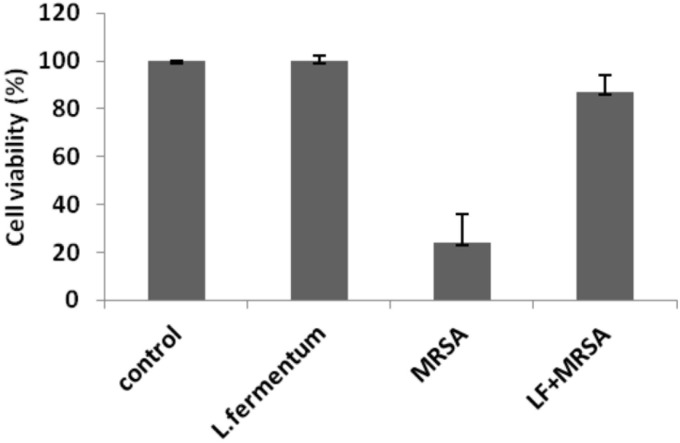
Cell viability of Caco-2 cells after the treatment with *L. fermentum*, MRSA and displacement experiment. Cell viability of Caco-2 cells was analyzed using MTT assay at 24 h of post-infection.

Effects of *L. fermentum* 8711 and MRSA on cell viability of Caco-2 cells were determined at 24 h of post-infection using MTT assay. *L. fermentum* did not affect the Caco-2 cells, and the viability was ∼99% similar to that of untreated cells. MRSA induced a significant cytotoxic effect in Caco-2 cells, and only 24% cells were viable after 24% of infection. When the MRSA infected Caco-2 cells were treated with *L. fermentum* (displacement assay), the cell viability was increased to a level of 86% (**Figure 6**). Thus, *L. fermentum* has reduced the MRSA-induced cytotoxicity.

### Diminished Cytotoxicity of MRSA in Caco-2 Cells Upon *L. fermentum* Treatment

**FIGURE 7:**
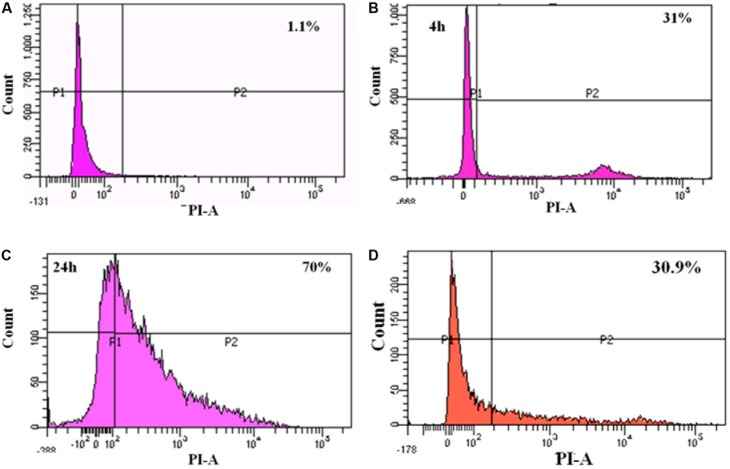
FACS analysis of dead cells: **(A)** un-infected Caco-2 cells; **(B)** 4 h post-infection with MRSA; **(C)** 24 h post-infection with MRSA; **(D)** 24 h post-infection with MRSA and *L. fermentum* (displacement experiment).

To study the cytotoxicity, Caco-2 cells infected with MRSA for various time points were subjected to live-dead assay using flow cytometry. At 4 h post-infection with MRSA, 31% of dead cells were identified, and after 24 h post-infection, dead cells were increased to 70% (**Figure 7**). *L. fermentum* also inhibited the cytotoxic effect of MRSA in Caco-2 cells. As shown in **Figure 7**, *L. fermentum* showed a protective effect against cytotoxicity induced by MRSA in Caco-2 cells. The dead cells were reduced to a level of 30.9% from 70%. This result suggests that *L. fermentum* effectively displaces the adhered MRSA from Caco-2 cells and further reduces the MRSA-induced cytotoxicity.

### Computational Prediction of Adhesins and Their Cellular Localization in *L. fermentum*

A total of 2266 protein sequences of *L. fermentum* MTCC 8711 were retrieved from NCBI, and the possible adhesins were predicted using SPAAN. Out of 2266 proteins, SPAAN predicted 98 proteins as putative adhesins or adhesin like proteins with Pad value > 0.7 (Supplementary Table S1). Of these, CELLO predicted 77 proteins to be localized in the extracellular space. Other 12 proteins were localized in the inner membrane and 9 proteins were cytoplasmically localized. Among the predicted adhesins, the majority of them were hypothetical proteins, and their roles are yet to be identified.

## Discussion

Lactic acid bacteria provide health benefits through various mechanisms. Adhesion of probiotic bacteria to the intestinal epithelium is one of the primary criteria for selecting a new probiotic strain (Alander et al., 1999). The adhesion properties can be evaluated from *in vitro* experiments with intestinal cell lines. Stable adhesion of probiotic bacteria may counteract the adhesion and invasion of pathogenic bacteria. The mechanisms of anti-adhesion properties of probiotics against pathogens include secretion of antimicrobial substances (e.g., organic acids, bacteriocin, or hydrogen peroxide), secretion of proteins that degrade carbohydrate receptors, and production of receptor analogs and biosurfactants (Vuotto et al., 2014). Several studies have reported the antagonistic effect of lactobacilli against various pathogens (Gueimonde et al., 2006; Li et al., 2011). Lactobacilli also stimulate the host immune system against pathogens (Coconnier et al., 1993; Schiffrin et al., 1995; Alander et al., 1999). The probiotic organisms can inhibit the adhesion of pathogens or displace the pathogens from intestinal cells (Presti et al., 2015). The inhibition or displacement of pathogens by probiotics could also be due to the competition for specific receptors (Bienenstock et al., 2013).

We examined inhibition, competition, and displacement assays to assess the ability of *L. fermentum* 8711 to inhibit the adherence of MRSA to Caco-2 cells. We found that *L. fermentum* significantly reduced the adherence of MRSA. Earlier, Ren et al. (2012) have reported similar levels of inhibition of adhesion of *S. aureus* to Caco-2 cells by another probiotic bacterium, *Lactobacillus salivaris*. Few other reports are also available demonstrating the ability of probiotic lactobacilli and bifidobacteria to inhibit the cell association and invasion by pathogenic bacteria (Bouchard et al., 2013; Presti et al., 2015). It is noteworthy that the inhibitory effect of *L. fermentum* on MRSA adhesion was significantly higher (96.5%) in displacement assay. Thus, the inhibition of MRSA adherence by *L. fermentum* may not be due to the competition for epithelial cell receptors. Other phenomena such as microbial antagonism by the antimicrobial substances produced by *L. fermentum* may also play a significant role in the displacement effect. Selection of probiotics that can displace a specific pathogen would be the logical therapeutic approach for the treatment of infections caused by gastroenteric pathogens.

In cytotoxicity assay, we have shown that *L. fermentum* was not cytotoxic, and also significantly reduced the MRSA-induced cytotoxicity on Caco-2 cells. Also, the morphology of the Caco-2 cells was not affected by the *L. fermentum* treatment. Thus, *L. fermentum* may also be involved in counteracting the cytotoxicity mechanisms exerted by the MRSA. For example, Maudsdotter et al. (2011) have reported that lactic acid secreted by lactobacilli can reduce epithelial cell damage caused by group A *Streptococcus* by degrading lipoteichoic acid. Banerjee et al. (2009) have reported that *Lactobacillus delbrueckii* can inhibit cytotoxic effects *Clostridium difficile* to Caco-2 cells by the secretion of antitoxic compounds. Therefore, the mechanisms of anti-adhesion and anti-cytotoxic effects of *L. fermentum* will be investigated further.

Earlier, the genome of *L. fermentum* 8711 was sequenced (Jayashree et al., 2013). Two genes coding for bile salt hydrolase were identified and characterized, which could lower the cholesterol levels in humans (Jayashree et al., 2014). Also, the genome contains a gene coding for colicin V synthesis protein and two genes coding for holin proteins. These proteins could be responsible for the better survivability in a competitive environment. In this study, we have identified 98 putative adhesins from the genome of *L. fermentum* 8711. Most of them are annotated as hypothetical proteins. Functional characterization of these proteins may help to understand the molecular mechanisms of adhesion of *L. fermentum* and their possible roles in the exclusion of pathogens from intestinal cells.

To summarize, we evaluated the adhesion property of *L. fermentum* 8711 on the human epithelial cells using Caco-2 cell line as a model. Also, we examined the anti-adhesion property of *L. fermentum* 8711 against MRSA in Caco-2 cells through inhibition, competition, and exclusion assays. We found that *L. fermentum* could efficiently adhere to the Caco-2 cells without any cytotoxic effect. Also, *L. fermentum* could efficiently inhibit, compete with and exclude the adhesion of MRSA on Caco-2 cells. Particularly, the inhibitory activity of *L. fermentum* on MRSA was significantly higher in exclusion assay than the adhesion and competition assays. Thus, *L. fermentum* has therapeutic potential to eliminate the MRSA from the gut epithelial cells. The molecular mechanisms of adhesion and anti-adhesion properties of *L. fermentum* should be studied further.

## Author Contributions

SJ, JeR, and PG designed the experiments. SJ, RK, SN, and JR did the experiments. SJ, RK, and JR wrote the paper.

## Conflict of Interest Statement

The authors declare that the research was conducted in the absence of any commercial or financial relationships that could be construed as a potential conflict of interest.
