# Supplementary material for: Anti-adhesion Property of the Potential Probiotic Strain Lactobacillus fermentum 8711 Against Methicillin-Resistant Staphylococcus aureus (MRSA)
Source: Front Microbiol. 2018 Mar 8;9:411. doi: 10.3389/fmicb.2018.00411 (PMC5852077; doi:10.3389/fmicb.2018.00411)
Supplement: Supplementary file 1 [file Table_1.DOC]

**Supplementary Table S1.** List of predicted adhesin-like proteins in *Lactobacillus fermentum* MTCC 8711 using SPAAN

| **S. No** | **Protein ID** | **Description** | **P_ad_ value*** | **Localization** |
| --- | --- | --- | --- | --- |
|  | EQC58873 | glycoside hydrolase family 25 | 0.937253 | Extracellular |
|  | EQC59736 | mannosyl-glycoprotein endo-beta-N-acetylglucosamidase | 0.933631 | Extracellular |
|  | EQC59472 | hypothetical protein N219_08410 | 0.927881 | Extracellular |
|  | EQC58807 | peptidoglycan-binding protein LysM | 0.927323 | Extracellular |
|  | EQC58524 | hypothetical protein N219_11320 | 0.920844 | Extracellular |
|  | EQC59796 | hypothetical protein N219_00875 | 0.919731 | Extracellular |
|  | EQC59452 | hypothetical protein N219_08135 | 0.9155 | Extracellular |
|  | EQC60344 | hypothetical protein N219_10915 | 0.910888 | Extracellular |
|  | EQC59864 | hypothetical protein N219_02465 | 0.906945 | Extracellular |
|  | EQC59903 | hypothetical protein N219_02905 | 0.905402 | Extracellular |
|  | EQC59955 | hypothetical protein N219_03985 | 0.902576 | Extracellular |
|  | EQC57678 | peptidase S8 (plasmid) | 0.894724 | Extracellular |
|  | EQC58802 | hypothetical protein N219_02245 | 0.885311 | Extracellular |
|  | EQC60295 | hypothetical protein N219_09970 | 0.878747 | Extracellular |
|  | EQC59391 | penicillin-binding protein | 0.873095 | Extracellular |
|  | EQC58648 | cell surface protein | 0.871942 | Extracellular |
|  | EQC57731 | adhesin (plasmid) | 0.865519 | Extracellular |
|  | EQC58479 | hypothetical protein N219_11610 | 0.86229 | Extracellular |
|  | EQC59439 | N-acetylmuramidase | 0.859205 | Extracellular |
|  | EQC59918 | hypothetical protein N219_03215 | 0.848373 | Extracellular |
|  | EQC59898 | hypothetical protein N219_02880 | 0.846321 | Extracellular |
|  | EQC58448 | hypothetical protein N219_11750 | 0.838187 | Extracellular |
|  | EQC57629 | hypothetical protein N219_13695 (plasmid) | 0.836265 | Extracellular |
|  | EQC59317 | penicillin-binding protein 1A | 0.835502 | Extracellular |
|  | EQC59618 | FMN-binding protein | 0.834054 | Extracellular |
|  | EQC58344 | hypothetical protein N219_11915 | 0.832817 | Extracellular |
|  | EQC59201 | DNA-entry nuclease | 0.831687 | Extracellular |
|  | EQC58674 | hypothetical protein N219_01160 | 0.827916 | Extracellular |
|  | EQC57682 | hypothetical protein N219_13480 (plasmid) | 0.825803 | Extracellular |
|  | EQC60284 | hypothetical protein N219_09820 | 0.824092 | Extracellular |
|  | EQC59838 | hypothetical protein N219_01485 | 0.823733 | Extracellular |
|  | EQC59795 | hypothetical protein N219_00870 | 0.82097 | Extracellular |
|  | EQC59509 | calcium-transporting ATPase | 0.817326 | Extracellular |
|  | EQC59821 | hypothetical protein N219_01360 | 0.816456 | Extracellular |
|  | EQC58523 | hypothetical protein N219_11315 | 0.815979 | Extracellular |
|  | EQC58661 | metal ABC transporter substrate-binding protein | 0.812768 | Inner Membrane |
|  | EQC59094 | ABC transporter substrate-binding protein | 0.809794 | Extracellular |
|  | EQC59495 | transcriptional regulator | 0.80691 | Extracellular |
|  | EQC58551 | single-stranded DNA-binding protein | 0.804809 | Extracellular |
|  | EQC58826 | peptidase M10A and M12B matrixin and adamalysin | 0.797161 | Extracellular |
|  | EQC60263 | hypothetical protein N219_09560 | 0.795311 | Extracellular |
|  | EQC57626 | hypothetical protein N219_13670 (plasmid) | 0.79384 | Extracellular |
|  | EQC59684 | ribokinase | 0.793758 | Cytoplasmic |
|  | EQC60197 | hypothetical protein N219_08190 | 0.792616 | Extracellular |
|  | EQC58522 | hypothetical protein N219_11310 | 0.789402 | Extracellular |
|  | EQC57803 | peptidoglycan hydrolase (plasmid) | 0.788018 | Extracellular |
|  | EQC59617 | ABC transporter permease | 0.785976 | Inner Membrane |
|  | EQC59653 | ribokinase | 0.781914 | Cytoplasmic |
|  | EQC58480 | hypothetical protein N219_11585 | 0.779151 | Extracellular |
|  | EQC58231 | hypothetical protein N219_12105 | 0.778327 | Inner Membrane |
|  | EQC57671 | hypothetical protein N219_13400 (plasmid) | 0.776634 | Extracellular |
|  | EQC59260 | peptide ABC transporter substrate-binding protein | 0.774188 | Inner Membrane |
|  | EQC59896 | hypothetical protein N219_02870 | 0.77349 | Extracellular |
|  | EQC58369 | hypothetical protein N219_11860 | 0.772557 | Extracellular |
|  | EQC57801 | thioredoxin (plasmid) | 0.771119 | Extracellular |
|  | EQC59811 | hypothetical protein N219_01035 | 0.77001 | Cytoplasmic |
|  | EQC59797 | hypothetical protein N219_00905 | 0.767017 | Extracellular |
|  | EQC60209 | hypothetical protein N219_08515 | 0.766712 | Extracellular |
|  | EQC60088 | hypothetical protein N219_06065 | 0.76628 | Extracellular |
|  | EQC57868 | beta-N-acetylglucosaminidase (plasmid) | 0.766187 | Inner Membrane |
|  | EQC57661 | beta-galactosidase (plasmid) | 0.7633 | Extracellular |
|  | EQC57820 | beta-galactosidase (plasmid) | 0.7633 | Extracellular |
|  | EQC57802 | hypothetical protein N219_13160 (plasmid) | 0.762625 | Extracellular |
|  | EQC60240 | hypothetical protein N219_09195 | 0.75837 | Extracellular |
|  | EQC60286 | hypothetical protein N219_09830 | 0.755314 | Extracellular |
|  | EQC58955 | competence protein | 0.753173 | Extracellular |
|  | EQC59997 | hypothetical protein N219_04670 | 0.746414 | Extracellular |
|  | EQC59897 | hypothetical protein N219_02875 | 0.745687 | Extracellular |
|  | EQC58977 | Inner Membrane protein | 0.745415 | Extracellular |
|  | EQC60024 | hypothetical protein N219_05060 | 0.745057 | Extracellular |
|  | EQC59302 | carbonate dehydratase | 0.743996 | Extracellular |
|  | EQC57826 | hypothetical protein N219_12695 (plasmid) | 0.743054 | Extracellular |
|  | EQC58711 | beta-galactosidase | 0.738164 | Cytoplasmic |
|  | EQC58928 | penicillin-binding protein 2B | 0.73599 | Inner Membrane |
|  | EQC58435 | hypothetical protein N219_11775 | 0.734233 | Extracellular |
|  | EQC59502 | metal-binding protein | 0.731776 | Cytoplasmic |
|  | EQC59596 | hypothetical protein N219_09520 | 0.730481 | Cytoplasmic |
|  | EQC59930 | cell division protein | 0.726823 | Inner Membrane |
|  | EQC60257 | sucrose-6-phosphate hydrolase | 0.726424 | Cytoplasmic |
|  | EQC57825 | peptidase (plasmid) | 0.724965 | Extracellular |
|  | EQC60294 | acetyltransferase | 0.722893 | Extracellular |
|  | EQC57917 | hypothetical protein N219_12530 (plasmid) | 0.721241 | Extracellular |
|  | EQC58528 | hypothetical protein N219_11290 | 0.720144 | Extracellular |
|  | EQC59426 | peptidylprolyl isomerase | 0.719007 | Extracellular |
|  | EQC59292 | homoserine dehydrogenase | 0.718059 | Cytoplasmic |
|  | EQC58938 | cysteine desulfurase | 0.715281 | Extracellular |
|  | EQC57694 | hypothetical protein N219_13575 (plasmid) | 0.714764 | Extracellular |
|  | EQC59798 | hypothetical protein N219_00910 | 0.714424 | Extracellular |
|  | EQC59209 | amino acid ABC transporter substrate-binding protein | 0.71288 | Inner Membrane |
|  | EQC59442 | carbohydrate kinase | 0.711638 | Cytoplasmic |
|  | EQC60084 | penicillin-binding protein | 0.71143 | Inner Membrane |
|  | EQC57928 | hypothetical protein N219_12595 (plasmid) | 0.71106 | Extracellular |
|  | EQC59894 | tape measure protein | 0.709769 | Extracellular |
|  | EQC59646 | peptide ABC transporter substrate-binding protein | 0.708862 | Inner Membrane |
|  | EQC59498 | Inner Membrane protein | 0.707924 | Inner Membrane |
|  | EQC60067 | hypothetical protein N219_05785 | 0.707675 | Inner Membrane |
|  | EQC59954 | hypothetical protein N219_03975 | 0.705497 | Extracellular |
|  | EQC60062 | hypothetical protein N219_05745 | 0.702079 | Extracellular |

^*^P_ad_ = (*P*_A_ ∗ fc_A_ +*P*_C_ ∗ fcC +*P*D ∗ fc_D_ +*P*_H_ ∗ fc_H_ +*P*_M_ ∗ fc_M_)/(fc_A_ + fc_C_ + fc_D_ + fc_H_ + fc_M_)

Where fci is the fraction of correlation of i-th module of the trained neural network, where i = A (Amino acid frequencies), C (Charge composition), D (Dipeptide frequencies), H (Hydrophobic composition) or M (Multiplet frequencies). The fractions of correlation fci represent the fractions of total entries that were predicted correctly (Pi, adhesin > 0.5 and Pi, non-adhesin < 0.5) (Sachdeva et al., 2005).
